# Acute radiation syndrome in a non-destructive testing worker: a case report

**DOI:** 10.1186/s40557-018-0270-8

**Published:** 2018-09-25

**Authors:** Ji-Sung Ahn, Jai-Dong Moon, Wonyang Kang, Hyeong-Min Lim, Seunghyeon Cho, Dae-Young Lim, Won-Ju Park

**Affiliations:** Department of Occupational and Environmental Medicine, Chonnam National University Hwasun Hospital, 322 Seoyang-ro, Hwasun-gun, Gwangju, Jeollanam-do 58128 Republic of Korea

**Keywords:** Acute radiation syndrome, Non-destructive testing, Pancytopenia, Radiation

## Abstract

**Background:**

In Korea, there were repeated radiation exposure accidents among non-destructive testing workers. Most of the cases involved local injury, such as radiation burns or hematopoietic cancer. Herein, we report a case of acute radiation syndrome caused by short periods of high exposure to ionizing radiation.

**Case presentation:**

In January 2017, Korea Information System on Occupational Exposure (KISOE) found that a 31-year-old man who had worked in a non-destructive testing company had been overexposed to radiation. The patient complained of symptoms of anorexia, general weakness, prostration, and mild dizziness for several days. He was anemic. The venous injection areas had bruises and bleeding tendency. Blood and bone marrow testing showed pancytopenia and the patient was diagnosed with acute radiation syndrome (white blood cells: 1400/cubic mm, hemoglobin: 7.1 g/dL, platelets: 14000/cubic mm). He was immediately prohibited from working and blood transfusion was commenced. The patient’s radiation exposure dose was over 1.4 Gy (95% confidence limits: 1.1–1.6) in lymphocyte depletion kinetics. It was revealed that the patient had been performing non-destructive tests without radiation shielding when working in high places of the large pipe surface.

**Conclusions:**

Exposure prevention is clearly possible in radiation-exposed workers. Strict legal amendments to safety procedures are essential to prevent repeated radiation exposure accidents.

## Background

In Korea, ionizing radiation is used in power plants, industries, medical and research fields. With the exception of nuclear power plants, it is most commonly used in the industrial sector, especially in non-destructive testing. Non-destructive inspection workers are continuously increasing to 5726 persons in 2009 and 7645 persons in 2015 [1]. Non-destructive testing is the use of radiation to identify defects in machinery, equipment, and piping. Using radiation, defects in welds on buildings and ships and joints in piping can be identified. Although this is a convenient method, there is always a risk of exposure to large amounts of radiation. In Korea, there have been repeated radiation overexposure accidents in non-destructive testing workers (Table 1). Most of the cases were local injuries such as radiation burns or hematopoietic cancer due to chronic or subchronic cumulative radiation exposure [2–14].

**Fig. 1:**
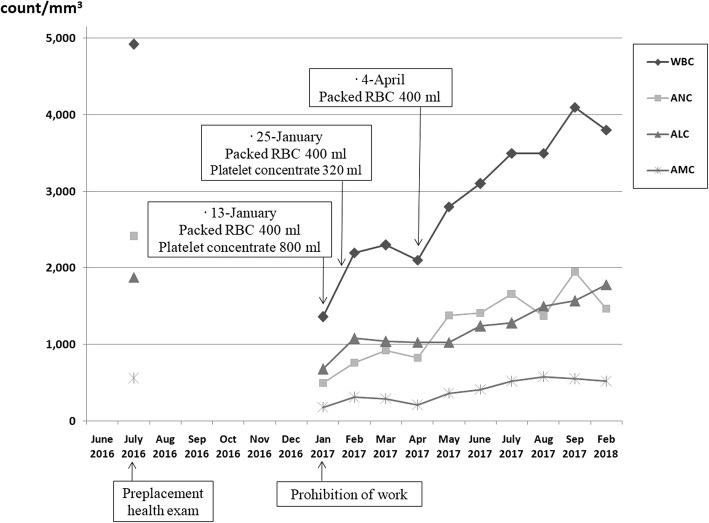
Changes in the laboratory results of the patient with acute radiation syndrome

**Table 1:** Case reports on accident exposure in Korean non-destructive testing workers

Year^a^	Sex	Disease	Age at diagnosis (years)	Working duration (years)	Reference
2017	Male	Acute radiation syndrome: pancytopenia, anemia	31	5	This study
2015	–	Radiodermatitis: ulcer at fingers	–	–	KINS [1]
2013	Male	Azoospermia	39	8	Park J, et al. [6]
2010	Male	Myelodysplastic syndrome	35	9	Oh MS, et al. [4]
2009	Male	Myelodysplastic syndrome	26	1	Oh MS, et al. [4]
2004	–	Radiodermatitis: necrosis at hand	–	–	KINS [11]
2003	–	Radiodermatitis: sclerosis at dorsum manus	25	–	KINS [11]
~ 2000	Male	Radiodermatitis: sclerosis at fingers	40	10	Park SW, et al. [10]
~ 2000	Male	Radiodermatitis: necrosis at palm	32	–	Park SW, et al. [10]
1999	–	Radiodermatitis: ulcer at fingers and palm	–	–	KINS [11]
1986~ 1996	Male	Radiodermatitis: ulcer at fingers	22	–	Kim KJ, et al. [5]
	Male	Radiodermatitis: ulcer at fingers	28	–	Kim KJ, et al. [5]
	Male	Radiodermatitis: edema, erythema at fingers	25	–	Kim KJ, et al. [5]
	Male	Radiodermatitis: erosion at palm	19	–	Kim KJ, et al. [5]
	Male	Radiodermatitis: ulcer, sclerosis at fingers	20	–	Kim KJ, et al. [5]
	Male	Radiodermatitis: ulcer, sclerosis at palm	21	–	Kim KJ, et al. [5]
~ 1990	Male	Radiodermatitis: desquamation, sclerosis at fingers	22	–	Ro YS [9]
1989	–	Amputation at fingers	–	–	KINS [11]
~ 1989	Male	Radiodermatitis: desquamation, sclerosis at fingers	22	–	Kim KJ, et al. [8]
	Male	Radiodermatitis: hardening at fingers	28	–	Kim KJ, et al. [8]
	Male	Radiodermatitis: hardening at fingers	25	–	Kim KJ, et al. [8]
	Male	Radiodermatitis: edema at palm	19	–	Kim KJ, et al. [8]
1984	Male	Radiodermatitis: soft tissue injury at palm	29	1	Yoon SC, et al. [7]
1983	Male	Radiodermatitis: elliptical ulcer at lower abdomen	29	2	Yoon SC, et al. [7]
1983	Male	Radiodermatitis: edema, amputation at finger	19	0.1	Yoon SC, et al. [7]

^a^Year of diagnosis

Acute radiation syndrome is a consequence of brief but heavy exposure (> 1 Gy) of all or part of the body to ionizing radiation. The radiation disrupts chemical bonds, which causes molecular excitation and free radical formation. Highly reactive free radicals react with other essential molecular structures such as nucleic acids and enzymes, which in turn disrupts cellular function. In particular, spermatogonia, lymphocytes, blast cells, other hematopoietic cells, small intestine, stomach, colon, epithelium and skin cells are radiation sensitive [15]. In Korea, cases of acute radiation syndrome caused by occupational exposure are very rare. And there were no cases reported previously. Herein we report a case of acute radiation syndrome caused by relatively short periods of high exposure to ionizing radiation and discuss the problems of the current system.

## Case presentation

### Patient

Thirty-one-year-old male.

### Chief complaint

Anorexia, general weakness, prostration, and mild dizziness for several days.

### Past medical history and family disease

No specific findings.

### Social history

Current smoker (15 pack-years) and social drinker.

### History of present illness

The patient had worked for 5 years in Yeosu National Industrial Complex as a non-destructive testing worker and had no job before. He performed radiographic testing using gamma radiation. In January 2017, the Korea Information System on Occupational Exposure (KISOE) found that his personal thermoluminescent dosimeter (TLD) badge indicated that he had exceeded the exposure limit. The patient’s TLD badge indicated that in December 2016, the patient’s radiation exposure dose for the month was 120 mSv. The patient’s radiation exposure dose was 1191 mSv according to the chromosome aberration test by South Korea’s Nuclear Safety and Security Commission (NSCC) [16]. The patient visited our hospital via the emergency room, and underwent a complete blood count test and bone marrow biopsy. It was revealed that the patient performed non-destructive tests without radiation shielding when working in high places of the large pipe surface.

### Physical examination

When the patient came to our hospital, he was clearly conscious with a blood pressure of 140/80 mmHg, temperature of 37.5 °C, pulse rate of 104 beats/min, and respiration rate of 20 breaths/min. He was anemic. The venous injection areas had bruises and bleeding tendency. There were no abnormal findings in the cornea and lens of the eye. We tried to perform semen analysis but failed due to the patient’s condition. Subsequently, semen analysis could not be performed because of refusal by the patient.

### Laboratory results

In a pre-placement medical examination conducted 6 months before the accident, all blood parameters were in the normal range: white blood cell count: 4920 cells/mm^3^, absolute neutrophil count: 2410 cells/mm^3^, absolute lymphocyte count: 1880 cells/mm^3^, hemoglobin: 14.7 g/dL, and platelet: 217 ×  10^3^/mm^3^. A blood test performed in the hospital after the symptoms appeared showed severe pancytopenia: white blood cell count: 1360 cells/mm^3^, absolute neutrophil count: 500 cells/mm^3^, absolute lymphocyte count: 680 cells/mm^3^, hemoglobin: 7.1 g/dL, and platelet: 14 × 10^3^/mm^3^. The laboratory results showed a slight recovery after 26 days from the date of prohibition of work: white blood cell count: 2200 cells/mm^3^, absolute neutrophil count: 760 cells/mm^3^, absolute lymphocyte count: 1080 cells/mm^3^. After a 13-month follow-up on the blood test, pancytopenia improved over time; however, it did not recover to the level before the accident (Table 2, Fig. 1).

**Table 2:** Changes in the laboratory results of the patient with acute radiation exposure

Variables	Normal ranges	Preplacement medical exam^a^	Days after prohibition of work							
			0	1	26	82	119	174	256	405
WBC (count/mm^3^)	4000-10,800	4920	1360	1400	2200	2100	2800	3500	4100	3800
ANC (count/mm^3^)	1500-8000	2410	500	590	760	830	1380	1660	1950	1470
ALC (count/mm^3^)	1500-4000	1880	680	650	1080	1030	1030	1280	1570	1780
AMC (count/mm^3^)	200–1000	560	179	150	310	210	360	520	550	520
Hemoglobin (g/dl)	12–18	14.7	7.5	7.1	10.3	7.3	10.8	13.2	14.0	14.1
RBC (× 10^3^/mm^3^)	420–610	458		201	293	191	284	370	407	419
Hematocrit (%)	37–52	44.4		19.4	28.9	20.7	31.3	38.8	41.4	42.8
Platelet (× 10^3^/mm^3^)	130–450	217		14	27	38	51	130	101	115
Reticulocyte (%)	0.5–1.5			1.05	1.72	1.69	1.9	1.54		

*Abbreviations: WBC* white blood cell, *ANC* absolute neutrophil count, *ALC* absolute lymphocyte count, *AMC* absolute monocyte count, *RBC* red blood cell

^a^The preplacement medical examination was conducted 6 months prior to the accident

### Assessment of radiation dose

The patient’s radiation exposure dose was assessed using lymphocyte depletion kinetics. The patient’s lowest absolute lymphocyte count was 0.65 × 10^9^ cells/L, and the radiation exposure dose based on this count was 1.4 Gy (95% confidence limits: 1.1–1.6) [17, 18]. The patient’s radiation exposure dose was 1191 mSv in the chromosome aberration test by South Korea’s NSCC [16].

## Discussion

On April 26, 1986, an explosion accident occurred in the Chernobyl nuclear power plant. On the day of the accident, about 600 employees worked in the site, and 134 of them had acute radiation syndrome due to the radiation exposure of 0.8 to 16 Gy. Within the first 3 months of the accident, 28 men died from radiation exposure [19]. In 1987, medical cesium-137 was stolen in Goiania, Brazil. Twenty villagers showed acute radiation syndrome, and 4 of them died [20, 21]. After these catastrophic accidents, the risk of radiation was spread widely; consequently, acute radiation syndrome cases have become rare due to strict controls. In the Fukushima nuclear power plant accident in March 2011, there was no report of acute radiation syndrome patients.

In Korea, radiation accidents repeatedly occurred to non-destructive testing workers, and tighter regulations and harsher punishment policies have been introduced. However, their fundamentally poor working conditions have not improved greatly as they still perform non-destructive testing overnight. To reduce radiation exposure doses in non-destructive testing workers, what needs to be done is: (i) use suitable shielding equipment, (ii) keep a safe distance from the source of radiation, (iii) reduce exposure time, and (iv) use safety equipment such as the personal dosimeter. In addition, replacing the penetration test that uses radiation with the ultrasound test could be a fundamental solution. While many applicable laws and rules are enforced for safety management, radiation exposure accidents have continued to occur in Korea. Furthermore, there could be many radiation over-exposure accidents that have not been reported. Along with further legislation, it is required to provide training and improve perception of changes among employers and employees.

A pre-placement medical examination is a process that conducts a medical check-up on the workers who are scheduled to do a job that exposes themselves to hazards before they are assigned to the job and identifies whether there is any health problem. A pre-placement medical examination was performed on this study’s subject 6 months before the accident pursuant to the Occupational Safety and Health Act. As a result, it was demonstrated from a blood test that all parameters were in the normal range and the subject was healthy before the accident. Accordingly, it served as definitive evidence proving that the subject’s pancytopenia was an acute health effect from the radiation exposure accident. This is a case where a pre-placement medical examination, which is mandatory, was effective for the healthcare of a worker. Had a pre-placement medical examination not been provided, it would have been difficult to identify whether the patient’s pancytopenia was a personal or occupational disease.

In Korea, workers exposed to health hazards receive a special health examination periodically, which is performed by occupational and environmental medicine specialists pursuant to the Occupational Safety and Health Act. However, a special health examination by occupational and environmental medicine specialists is not necessarily provided to radiation-exposed workers if a general health examination was provided already as per the Nuclear Safety Act or the Regulation on the Safety Management of Diagnostic Radiation Equipment. Occupational and environmental medicine specialists are medical doctors specializing in the prevention and early diagnosis of occupational diseases. A strict special health examination by occupational and environmental medicine specialists is required periodically for radiation-exposed workers just as for other workers exposed to health hazards.

## Conclusions

Exposure prevention is clearly possible in radiation-exposed workers. Strict legal amendments are essential to prevent repeated radiation exposure accidents. Health care of radiation-exposed workers should be strictly managed by occupational medicine specialists.

## Abbreviations

ALC: Absolute lymphocyte count
AMC: Absolute monocyte count
ANC: Absolute neutrophil count
WBC: White blood cell
